# Supplementary material for: Modeling glioblastoma heterogeneity as a dynamic network of cell states
Source: Mol Syst Biol. 2021 Sep 16;17(9):e10105. doi: 10.15252/msb.202010105 (PMC8444284; doi:10.15252/msb.202010105)
Supplement: Supplementary file 6 — Source Data for Figure 5 [file MSB-17-e10105-s004.zip › Figure5A_sourcedata/GSEA_3071/hallmarks_stateIV.GseaPreranked.1623742551702/HALLMARK_INFLAMMATORY_RESPONSE.html]

Details for gene set HALLMARK\_INFLAMMATORY\_RESPONSE[GSEA]

|  || Dataset | state53071 |
| Phenotype | NoPhenotypeAvailable |
| Upregulated in class | na\_neg |
| GeneSet | HALLMARK\_INFLAMMATORY\_RESPONSE |
| Enrichment Score (ES) | -0.43598312 |
| Normalized Enrichment Score (NES) | -2.1026895 |
| Nominal p-value | 0.0 |
| FDR q-value | 3.6145782E-4 |
| FWER p-Value | 0.003 |
Table: GSEA Results Summary

  

Fig 1: Enrichment plot: HALLMARK\_INFLAMMATORY\_RESPONSE      
 Profile of the Running ES Score & Positions of GeneSet Members on the Rank Ordered List

  

| PROBE | GENE SYMBOL | GENE\_TITLE | RANK IN GENE LIST | RANK METRIC SCORE | RUNNING ES | CORE ENRICHMENT || 1 | SLC1A2 |  |  | 48 | 1.860 | 0.0236 | No |
| 2 | PDPN |  |  | 76 | 1.689 | 0.0482 | No |
| 3 | BTG2 |  |  | 200 | 1.260 | 0.0469 | No |
| 4 | CD82 |  |  | 223 | 1.202 | 0.0639 | No |
| 5 | RGS16 |  |  | 237 | 1.178 | 0.0822 | No |
| 6 | SRI |  |  | 298 | 1.037 | 0.0890 | No |
| 7 | ITGB8 |  |  | 307 | 1.027 | 0.1056 | No |
| 8 | CALCRL |  |  | 362 | 0.953 | 0.1120 | No |
| 9 | PDE4B |  |  | 519 | 0.798 | 0.0963 | No |
| 10 | HRH1 |  |  | 1023 | 0.513 | 0.0090 | No |
| 11 | HAS2 |  |  | 1066 | 0.502 | 0.0099 | No |
| 12 | KIF1B |  |  | 1143 | 0.482 | 0.0038 | No |
| 13 | CSF1 |  |  | 1189 | 0.469 | 0.0035 | No |
| 14 | RAF1 |  |  | 1296 | 0.441 | -0.0090 | No |
| 15 | RASGRP1 |  |  | 1384 | 0.420 | -0.0183 | No |
| 16 | CMKLR1 |  |  | 1705 | 0.349 | -0.0734 | No |
| 17 | KCNJ2 |  |  | 1722 | 0.346 | -0.0703 | No |
| 18 | PTGER4 |  |  | 1797 | 0.331 | -0.0787 | No |
| 19 | FZD5 |  |  | 2089 | 0.285 | -0.1293 | No |
| 20 | PTPRE |  |  | 2187 | 0.269 | -0.1431 | No |
| 21 | SLC4A4 |  |  | 2227 | 0.265 | -0.1459 | No |
| 22 | IL15RA |  |  | 2354 | -0.253 | -0.1656 | No |
| 23 | PVR |  |  | 2496 | -0.269 | -0.1878 | No |
| 24 | NFKBIA |  |  | 2721 | -0.296 | -0.2255 | No |
| 25 | ITGB3 |  |  | 2905 | -0.319 | -0.2549 | No |
| 26 | ABCA1 |  |  | 3078 | -0.342 | -0.2818 | No |
| 27 | HIF1A |  |  | 3542 | -0.418 | -0.3630 | No |
| 28 | PCDH7 |  |  | 3576 | -0.423 | -0.3619 | No |
| 29 | TLR3 |  |  | 3642 | -0.435 | -0.3666 | No |
| 30 | RHOG |  |  | 3811 | -0.469 | -0.3905 | No |
| 31 | ITGA5 |  |  | 3864 | -0.480 | -0.3920 | No |
| 32 | HBEGF |  |  | 3905 | -0.488 | -0.3911 | No |
| 33 | GNAI3 |  |  | 3945 | -0.496 | -0.3898 | No |
| 34 | NFKB1 |  |  | 3989 | -0.507 | -0.3891 | No |
| 35 | DCBLD2 |  |  | 4038 | -0.521 | -0.3891 | No |
| 36 | SGMS2 |  |  | 4069 | -0.531 | -0.3854 | No |
| 37 | RIPK2 |  |  | 4107 | -0.542 | -0.3830 | No |
| 38 | IL15 |  |  | 4186 | -0.564 | -0.3880 | No |
| 39 | TNFSF10 |  |  | 4229 | -0.574 | -0.3859 | No |
| 40 | ATP2B1 |  |  | 4385 | -0.631 | -0.4044 | No |
| 41 | NAMPT |  |  | 4551 | -0.705 | -0.4235 | Yes |
| 42 | ADORA2B |  |  | 4590 | -0.729 | -0.4180 | Yes |
| 43 | SLC7A1 |  |  | 4614 | -0.740 | -0.4093 | Yes |
| 44 | ADRM1 |  |  | 4636 | -0.759 | -0.3999 | Yes |
| 45 | CD55 |  |  | 4794 | -0.857 | -0.4149 | Yes |
| 46 | LIF |  |  | 4841 | -0.907 | -0.4077 | Yes |
| 47 | MYC |  |  | 4883 | -0.947 | -0.3988 | Yes |
| 48 | KLF6 |  |  | 4953 | -1.024 | -0.3940 | Yes |
| 49 | EMP3 |  |  | 4960 | -1.029 | -0.3770 | Yes |
| 50 | LPAR1 |  |  | 4963 | -1.034 | -0.3591 | Yes |
| 51 | MMP14 |  |  | 4983 | -1.064 | -0.3440 | Yes |
| 52 | NMI |  |  | 4984 | -1.065 | -0.3252 | Yes |
| 53 | TNFAIP6 |  |  | 4987 | -1.072 | -0.3067 | Yes |
| 54 | CD70 |  |  | 5024 | -1.136 | -0.2936 | Yes |
| 55 | SPHK1 |  |  | 5040 | -1.169 | -0.2758 | Yes |
| 56 | EDN1 |  |  | 5081 | -1.246 | -0.2615 | Yes |
| 57 | TIMP1 |  |  | 5086 | -1.257 | -0.2401 | Yes |
| 58 | IRF1 |  |  | 5126 | -1.345 | -0.2238 | Yes |
| 59 | MET |  |  | 5153 | -1.438 | -0.2035 | Yes |
| 60 | SERPINE1 |  |  | 5162 | -1.501 | -0.1785 | Yes |
| 61 | AXL |  |  | 5165 | -1.518 | -0.1521 | Yes |
| 62 | PLAUR |  |  | 5213 | -1.721 | -0.1308 | Yes |
| 63 | TPBG |  |  | 5221 | -1.800 | -0.1004 | Yes |
| 64 | IL18 |  |  | 5224 | -1.814 | -0.0688 | Yes |
| 65 | F3 |  |  | 5230 | -1.856 | -0.0370 | Yes |
| 66 | INHBA |  |  | 5280 | -2.740 | 0.0019 | Yes |
Table: GSEA details [plain text format]

  

Fig 2: HALLMARK\_INFLAMMATORY\_RESPONSE: Random ES distribution      
 Gene set null distribution of ES for **HALLMARK\_INFLAMMATORY\_RESPONSE**
